# Unraveling the Complexities of Mast Cells in Health and Disease

**DOI:** 10.3390/ijms25073791

**Published:** 2024-03-28

**Authors:** Davide Firinu

**Affiliations:** Department of Medical Sciences and Public Health, University of Cagliari, 09124 Cagliari, Italy; davidefirinu@yahoo.it

As we draw the curtain on this Special Issue dedicated to the intricate roles of mast cells (MCs) in health and disease, we reflect on the insights garnered from the array of research articles featured within the published papers of the *International Journal of Molecular Sciences* (*IJMS*). Each manuscript contributes significantly to our evolving comprehension of MC biology and its implications across various pathological contexts.

The investigation conducted by Tsukada et al. [1] presents a compelling correlation between elevated HbA1c levels and the phenotypic characteristics of mast cells within the infrapatellar fat pad of patients afflicted with knee osteoarthritis (KOA). This exploration into the intricate interplay between diabetes mellitus (DM) and KOA pathogenesis underscores the nuanced molecular mechanisms linking metabolic dysregulation to inflammatory processes mediated by mast cells within the joint microenvironment.

Similarly, the study by Rijavec et al. [2] delves into the enigmatic landscape of mast cell disorders, shedding light on the potential implications of mastocytosis in a series of fatal hymenoptera-venom-triggered anaphylaxis. Through examination of clinical data and mast cell clonality assays, this study underscores the importance of recognizing and managing clonal mast cell disorders in the context of severe allergic reactions, thus advocating for improved diagnostic and therapeutic strategies.

Furthermore, the exploration undertaken by Serrao et al. [3] provides novel insights into the pathogenesis of systemic mastocytosis, unveiling the interactome of salivary cystatin D in patients afflicted with this rare clonal disorder. By elucidating the intricate molecular interactions underlying mast cell dysregulation, this study not only advances our understanding of mastocytosis but also identifies potential preliminary salivary biomarkers for disease diagnosis.

In a complementary vein, the review authored by Costanzo et al. [4] offers a comprehensive overview of mast cells’ pivotal roles in upper and lower airway diseases. By synthesizing contemporary knowledge on MC biology and their contributions to allergic inflammation, infection response, and tissue homeostasis, this review underscores the multifaceted functions of mast cells as sentinels at the forefront of immune defense.

Lastly, the review performed by Parente et al. [5] delves into the intricate landscape of mast cell activation and proliferation, unveiling a plethora of secretory and membrane-associated biomarkers indicative of MC dysregulation. By elucidating the molecular signatures of MC-related disorders, this manuscript offers insights into the diagnosis and therapeutic targeting of aberrant mast cell responses in allergic and inflammatory diseases.

Additionally, previous studies have revealed that olfactory function is impaired in patients diagnosed with mastocytosis, as demonstrated by Masala et al. [6], but the pathomechanisms are yet to be demonstrated. Furthermore, Serrao et al. [7] utilized top-down proteomics to uncover significant variations in the protein profile of human saliva in individuals afflicted with mastocytosis, underscoring the potential of salivary biomarkers in disease detection and monitoring. Therefore, there is a need to replicate such a study and to find actual validation of the proposed biomarkers or clinical findings reported.

We express our profound appreciation to the authors, reviewers, and editorial team whose dedication and scholarly contributions have enriched our understanding of mast cell biology and its profound implications in immunity and disease. May the knowledge disseminated within these manuscripts serve as a beacon guiding future research endeavors aimed at unraveling the complexities of mast cell biology and translating these insights into innovative therapeutic strategies for the benefit of patients.
